# A Concise Route for the Synthesis of Tetracyclic Meroterpenoids: (±)-Aureol Preparation and Mechanistic Interpretation

**DOI:** 10.3390/md18090441

**Published:** 2020-08-26

**Authors:** Antonio Rosales Martínez, Lourdes Enríquez, Martín Jaraíz, Laura Pozo Morales, Ignacio Rodríguez-García, Emilio Díaz Ojeda

**Affiliations:** 1 Department of Chemical Engineering, Escuela Politécnica Superior, University of Sevilla, 41011 Sevilla, Spain; lauratar@us.es (L.P.M.); emidi@us.es (E.D.O.); 2 Department of Electricity and Electronics, University of Valladolid, 47011 Valladolid, Spain; louenr@tel.uva.es (L.E.); mjaraiz@ele.uva.es (M.J.); 3 Organic Chemistry, CeiA3, University of Almería, 04120 Almería, Spain; irodrigu@ual.es

**Keywords:** aureol, tetracyclic meroterpenoids, natural products synthesis

## Abstract

A new concise general methodology for the synthesis of different tetracyclic meroterpenoids is reported: (±)-aureol (**1**), the key intermediate of this general route. The synthesis of (±)-aureol (**1**) was achieved in seven steps (28% overall yield) from (±)-albicanol. The key steps of this route include a C–C bond-forming reaction between (±)-albicanal and a lithiated arene unit and a rearrangement involving 1,2-hydride and 1,2-methyl shifts promoted by BF_3_•Et_2_O as activator and water as initiator.

## 1. Introduction

**Figure 1:**
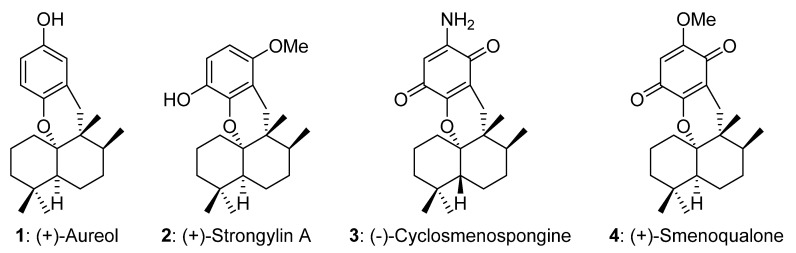
Selected members of tetracyclic meroterpenoids.

Marine sponges appear to have become an almost inexhaustible source of new natural compounds, showing a broad spectrum of biological activities and different structural patterns. Among these compounds there is a structurally unique class of natural products, the meroterpenoids, which are constituted by a sesquiterpene unit linked to a phenolic or quinone moiety [1]. Important examples of tetracyclic meroterpenoids (Figure 1) include (+)-aureol (**1**) [2,3], (+)-strongylin A (**2**) [4], (-)-cyclosmenospongine (**3**) [5] and (+)-smenoqualone (**4**) [6].

(+)-Aureol (**1**) was initially isolated and characterized by Faulker et al. [2] from the Caribbean sponge *Smeonspongia aurea*. It was later also found in some other species of Caribbean sponges, *Verongula gigantea* and *Smenospongia* sp. [7]. (+)-Aureol (**1**) is a tetracyclic meroterpenoid with a unique structure that combines a *cis*-decalin system with a substituted benzopyran moiety. It shows anti-influenza-A virus activity [8] and selective cytotoxicity against human tumor cells, including colon adenocarcinoma HT-29 cells [9] and nonsmall cell lung cancer A549 [9].

Although the tetracyclic meroterpenoids have exclusive structural features and a wide assortment of biological activities, only one highly modular and robust platform for the synthesis of this class of natural products has been reported to date [10]. The rest of the reported routes are synthetic operations (10–27 linear steps) that have not enabled straightforward access to the whole family of these interesting natural products [11,12,13,14,15,16,17,18,19,20].

## 2. Results and Discussion

As a continuation of our research on the synthesis of marine natural bioactive compounds [18,21,22,23], we have developed a new concise route for the synthesis of tetracyclic meroterpenoids. In this new synthetic route, aureol (**1**) is the key intermediate from which other tetracyclic meroterpenoids, such as **2**, **3** and **4**, can be easily synthesized by simple functional modification of its aromatic ring.

**Scheme 1:**
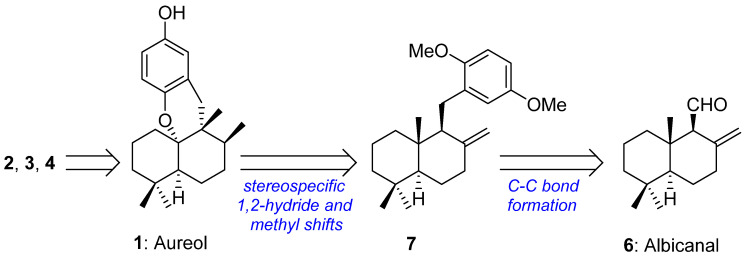
Retrosynthesis of tetracyclic meroterpenoids.

We thought the synthesis of **1** could be achieved through a coupling of albicanal (**6**) with 2-lithiohydroquinone dimethyl ether and a biogenetic-type rearrangement (previously explored by us) as pivotal steps (Scheme 1).

**Scheme 2:**
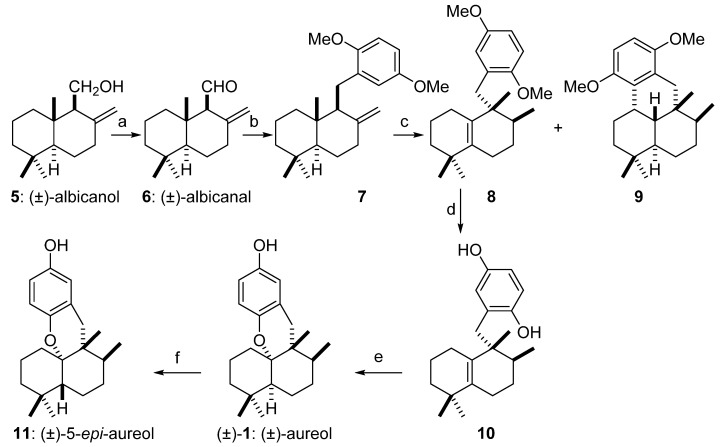
*Reagents and conditions:* (**a**) Dess–Martin, 99.7%; (**b**) (i) Hydroquinone dimethyl ether (3 equiv), Et_2_O, *sec*-BuLi (2 equiv), 5 min at 0 °C, 3 h at room temperature (rt). Then, **6** (1 equiv), Et_2_O, 5 min, rt, quantitative; (ii) Liquid NH_3_, THF, Li (5.3 equiv), 15 min, −78 °C. Then, mixture of benzylalcohols (1 equiv), THF, 15 min, −78 °C. Finally, NH_4_Cl (13.6 equiv), 30 min, −78 °C, 90% (two steps); (**c**) **7** (1 equiv), BF_3_•Et_2_O (5.0 equiv), CH_2_Cl_2_, 5 h, −50 to −5 °C, 62% (**8**), 28% (**9**); (**d**) (i) **8** (1 equiv), AgO (2.0 equiv), 6N HNO_3_ (3.0 equiv), 1,4-dioxane, rt, 15 min; (ii) 10% Pd/C (0.05 equiv), H_2_ (1 atm), CHCl_3_, 25 min, rt, 82%; (**e**) **10** (1 equiv), BF_3_•Et_2_O (4.5 equiv), CH_2_Cl_2_, −60 to −20 °C, 3 h, 62%; (**f**) HI, benzene, 90 °C, ref. 10, 87%.

The synthesis of (±)-aureol ((±)-**1**) (Scheme 2) used as starting material (±)-albicanol (**5**), which was prepared through Cp_2_TiCl-catalyzed radical cascade cyclization of epoxy-farnesyl acetate, as previously reported by us and others [24,25]. Dess–Martin oxidation of **5** almost quantitatively afforded (±)-albicanal (**6**). The first key step was the coupling of (±)-albicanal (**6**) with the lithiated arene unit. For this purpose, an efficient and economical methodology previously reported by Seifert et al. [26] was used. In our hands, the addition of 2-lithiohydroquinone dimethyl ether to (±)-albicanal (**6**) gave a mixture of diastereomeric benzylic alcohols. In order to remove the free hydroxy group, the reaction crude was treated with lithium in liquid NH_3_/THF followed by NH_4_Cl. In this way, *trans*-decaline **7** was obtained in 90% yield (two steps).

The second key step in our synthesis of (±)-aureol ((±)-**1**) was based on a biogenetic-type rearrangement of **7** to give **8** that was previously reported by us [18]. In this way, a BF_3_•Et_2_O-mediated rearrangement of **7** leads to the formation of the desired product **8** as a single stereoisomer in a 62% yield, together with a minor tetracyclic compound **9** in a 28% yield. Demethylation of **8** following the conditions reported by Wright et al. [27] in the synthesis of natural compound (+)-frondosin gave **10** in an 82% yield over the two steps. Finally, cyclization of phenolic compound **10** was carried out with BF_3_•Et_2_O. This reaction afforded (±)-aureol ((±)-**1**) in a 62% yield. Physical and spectroscopic properties of synthetic (±)-aureol ((±)-**1**) matched those previously reported for the natural compound [2]. Thus, the synthesis of (±)-aureol ((±)-**1**) from (±)-albicanol (**5**) was completed in only seven steps and a global 28% yield, substantially improving the synthetic procedures previously published [11,12,13,14,15,16,17,18,19,20]. Moreover, a simple epimerization of aureol (**1**) to 5-*epi*-aureol (**11**) has already been reported [10]. From these two compounds, aureol (**1**) and 5-*epi*-aureol (**11**), adequate functionalization sequences can lead to (-)-cyclomenospongine (**3**), (+)-strongylin A (**2**) and (+)-smenoquealone (**4**), sequences that can be considered alternative formal syntheses of these tetracyclic compounds [9,28]. In this way, the methodology here described can be considered a general method for the synthesis of tetracyclic meroterponoids.

**Scheme 3:**
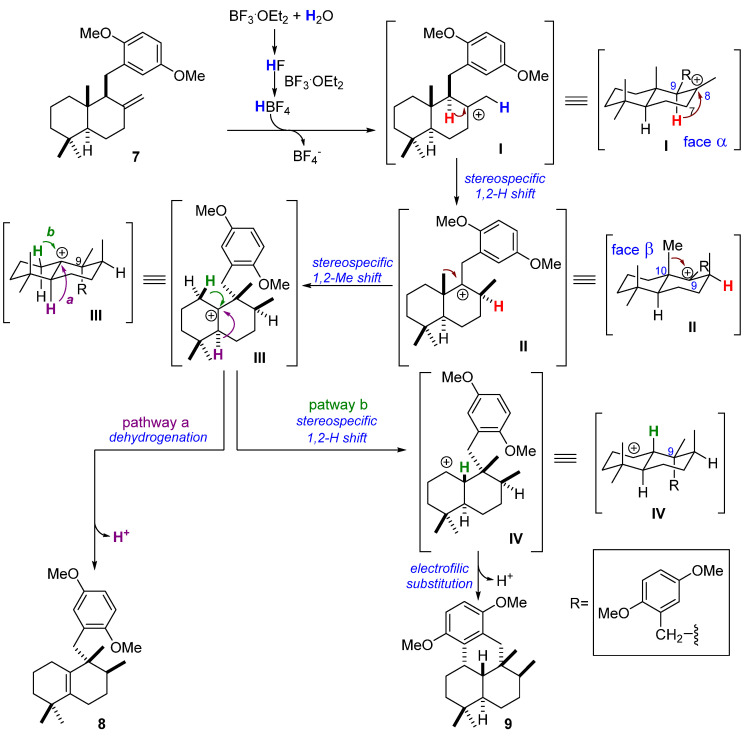
Proposed reaction mechanisms for the formation of tetrasubstituted alkene **8** and by-product **9**.

The transformation of the exocyclic alkene **7** into the rearranged products **8** and **9** can be rationalized as depicted in Scheme 3. It is known that pure Lewis acids, such as boron trifluoride, are not effective initiators in alkene cationic polymerization [29], which makes more likely a pathway involving a proton transfer. On the other hand, it is well known that BF_3_•Et_2_O is very moisture-sensitive, and inevitably over time the HF that forms from the hydrolysis of BF_3_ will react with excess BF_3_ to form HBF_4_, which is a strong acid and possibly triggers the cationic rearrangement. Thus, when the exocyclic alkene group in the bicyclic compound **7** is activated by a proton, the tertiary carbocation intermediate **I** is formed. Since the cleavage of a C–H bond is usually easier than a C–C bond, the hydrogen on C9 has a higher migratory aptitude than the alkyl group. In addition, migration of any of the hydrogens on C7 would lead to a secondary carbocation, less stable. In this way, the carbocationic intermediate **II** would be formed. From the stereochemical point of view, the configuration of C9 facilitates a 1,2-hydrogen shift on the α-face of the carbocation intermediate **I** to form carbocation intermediate **II**. Subsequently, the configuration of C10 facilitates a 1,2-methyl shift on the β-face of the carbocation intermediate **II** to form the carbocation intermediate **III**, which leads (pathway a, Scheme 3), after losing a H^+^, to the major compound **8**. On the other hand, the intermediate **III** could suffer a 1,2-hydride shift from the C1 position to the carbocation on C10 to form the carbocationic intermediate **IV** (pathway b, Scheme 3), which can react with the aromatic ring by electrophilic substitution to generate the minor tetracyclic by-product **9**. In both pathways, a H^+^ is liberated, which can react with more alkene **7** to continue the catalytic cycle. On the other hand, the simultaneous formation of **8** and **9** suggests that the all of the abovementioned rearrangements leading from **7** to **8** are not part of a concerted process, but proceed through a series of rapidly interconverting carbocations.

## 3. Experimental Section

### 3.1. General Methods

All reagents were used as received from commercial sources. All solvents were distilled before use. THF was refluxed over Na and CH_2_Cl_2_ over calcium hydride before being distilled under an Ar atmosphere. Reaction products were purified by conventional column chromatography on Merck silica gel 50. Analytical thin-layer chromatography (TLC) was performed on 0.2 mm DC-Fertigfolien Alugram^®^ Xtra Sil G/UV254 silica gel plates and visualized under a UV lamp or by immersion in an ethanol solution of phosphomolybdic acid (7%) followed by heating. ^1^H and ^13^C NMR spectra were recorded in Varian spectrometers operating at 300, 500 or 600 MHz. CDCl_3_ was always used as NMR solvent. (±)-Albicanol was prepared from commercial farnesol according to a known procedure [22,24]. Copies of ^1^H and ^13^C NMR spectra of relevant known compounds are provided in Supplementary Materials.

### 3.2. Dess–Martin Oxidation of (±)-Albicanol ***5***

To a CH_2_Cl_2_ (35 mL) solution of compound **5** (1.85 g, 8.32 mmol), 5.3 g of Dess–Martin periodinane (12.5 mmol) was added and the mixture stirred for 1 h at room temperature until completion by TLC. The mixture was then washed with NaHCO_3_ (sat. soln. 3 × 20 mL) and the organic phase dried over MgSO_4_, filtered and the solvent removed in vacuo. Chromatographic purification of the crude residue (silica gel column, Hexane/AcOEt 9:1) yielded (±)-albicanal (**6**) (1.83 g, 8.30 mmol, 99.7%) as a colorless oil. ^1^H and ^13^C NMR were identical to those previously reported [30].

### 3.3. Synthesis of Cis-Decaline ***7***

Hydroquinone dimethyl ether (0.83 g, 6.0 mmol) was dissolved in Et_2_O (13 mL) and *sec*-BuLi (3.1 mL, 1.3 M in cyclohexane) was added at 0 °C. After stirring the mixture for 3 h at room temperature, a solution of (±)-albicanal (**6**) (440 mg, 2.0 mmol) in Et_2_O (3 mL) was dropwise added. The reaction was stirred for 5 min before dropwise addition of NH_4_Cl (0.3 mL of saturated solution). To the mixture was then added 3 mL of saturated NaCl-solution, the organic phase dried over anhydrous Na_2_SO_4_ and the solvent removed in vacuo.

A mixture of liquid NH_3_ (24 mL), THF (13 mL) and Li (70 mg, 10 mmol, granulate, Merck) at −78 °C was prepared and stirred for 15 min. To this mixture was added a solution of the former reaction crude in THF (7 mL). The reaction was then stirred for 15 min at the same temperature. After that, NH_4_Cl (1.4 g) was added in portions (a change in color was observed from dark blue to colorless). Next, the mixture was allowed to reach room temperature to allow the evaporation of NH_3_ (2 h) and finally the reaction mixture was extracted with EtOAc. The combined organic layers were washed with brine, dried (anhydrous Na_2_SO_4_) and the solvent removed in vacuo. Column chromatography (Hexane/AcOEt 9:1) of the residue yielded the coupling product **7** (618 mg, 1.8 mmol) (90%), isolated as a colorless solid, m.p. 74–75 °C. IR (ATR) *v* (cm^−1^) 3000, 2940, 2860, 2830, 1640, 1605, 1495, 1460, 1440, 1210, 1050. ^1^H NMR (500 MHz, CDCl_3_) δ (ppm) 6.75–6.60 (m, 3H), 4.74 (s, 1H), 4.61 (s, 1H), 3.79 (s, 3H), 3.74 (s, 3H), 2.75 (d, *J* = 15 Hz, 2H), 2.36 (m, 1H), 2.22 (m, 1H), 2.01 (m, 1H), 1.88 (m, 1H), 1.80–1.20 (m, 9H), 0.90 (s, 3H), 0.84 (s, 3H), 0.82 (s, 3H). ^13^C NMR (125 MHz, CDCl_3_) δ (ppm) 153.2 (C), 151.7 (C), 148.3 (C),132.1 (C), 116,2 (CH), 110.8 (CH), 109.6 (CH), 107.6 (CH_2_), 55.9 (CH), 55.8 (CH_3_), 55.7 (CH), 55.5 (CH_3_), 42.2 (CH_2_), 39.9 (C), 39.1 (CH_2_), 38.3 (CH_2_), 33.6 (C), 33.6 (CH_3_), 24.4 (CH_2_), 23.2 (CH_2_), 21.8 (CH_3_), 19.5 (CH_2_), 14.6 (CH_3_). HRMS (ESI/Q-TOF) *m/z*: [M + H]^+^ calcd for C_23_H_35_O_2_ 343.2632; found 343.2629. ^1^H and ^13^C NMR data match with those previously reported [26].

### 3.4. Synthesis of Tetrasubstituted Olefin ***8***

BF_3_•Et_2_O (0.35 mL, 2.5 mmol) was added to a chilled solution (−50 °C) of **7** (171 mg, 0.5 mmol) in CH_2_Cl_2_ (50 mL). The mixture was slowly warmed up to −5 °C and stirred for 5 h. Then, the solvent was removed and the residue suspended in Et_2_O. The solution was washed with brine, dried over Na_2_SO_4_ and the solvent was removed in vacuo. Column chromatography of the residue (cyclohexane) yielded **8** (106 mg, 0.31 mmol, 62%) together with the by-product **9** (48 mg, 0.14 mmol, 28%). Compound **8** as a white solid; m.p. 58–61 °C.

IR (ATR) *v* (cm^−1^) 3020, 2930, 2850, 1620, 1592, 1495, 1240. ^1^H NMR (500 MHz, CDCl_3_) δ (ppm) 6.87 (d, *J* = 3 Hz, 1H), 6.75 (d, *J* = 9 Hz, 1H), 6.68 (dd, *J* = 9, 3.1 Hz, 1H), 3.76 (s, 3H), 3.73 (s, 3H), 2.93 (d, *J* = 15 Hz, 1H), 2.62 (d, *J* = 15 Hz, 1H), 2.09–2.01 (m, 4H), 1.96–1.90 (m, 1H), 1.69–1.58 (m, 4H), 1.39–1.32 (m, 2H), 1.01 (s, 3H), 1.00 (s, 3H), 0.92 (s, 3H), 0.79 (d, *J* = 7 Hz, 3H). ^13^C NMR (125 MHz, CDCl_3_) δ (ppm) 152.9 (C), 152.2 (C), 135.6 (C), 132.6 (C), 129.6 (C), 116.4 (CH), 110.8 (CH), 110.7 (CH), 55.7 (CH_3_), 55.5 (CH_3_), 41.4 (C), 39.7 (CH_2_), 34.5 (CH_2_), 34.2 (C), 33.3 (CH), 28.2 (CH_3_), 28.0 (CH_3_), 26.6 (CH_2_), 26.2 (CH_2_), 23.4 (CH_2_), 21.9 (CH_3_), 19.8 (CH_2_), 15.9 (CH_3_). HRMS (ESI/Q-TOF) *m/z*: [M + H]^+^ calcd for C_23_H_35_O_2_ 343.2632; found 343.2630. Compound **9** as a colorless solid, m.p. 111–113 °C. IR (ATR) *v* (cm^−1^) 3010, 2950, 2870, 1610, 1592, 1461, 1249. ^1^H NMR (500 MHz, CDCl_3_) δ (ppm) 6.67–6.63 (m, 2H), 3.79 (s, 3H), 3.74 (s, 3H), 3.08–3.00 (m, 3H), 2.12–2.06 (m, 2H), 1.60–1.10 (m, 9H), 1.01 (d, *J* = 13 Hz, 3H), 0.85 (s, 3H), 0.79 (s, 3H), 0.75 (s, 3H). ^13^C NMR (125 MHz, CDCl_3_) δ (ppm) 153.1 (C), 151.8 (C), 128.3 (C), 128.2 (C), 108.4 (CH), 106.5 (CH), 55.7 (CH_3_), 55.4 (CH_3_), 42.3 (CH), 39.0 (CH), 38.3 (CH), 38.1 (CH_2_), 34.5 (C), 33.5 (CH), 32.7 (CH_2_), 32.5 (C), 30.5 (CH_3_), 28.9 (CH_2_), 25.0 (CH_3_), 24.0 (CH_2_), 21.5 (CH_2_), 20.3 (CH_3_), 14.6 (CH_3_). HRMS (ESI/Q-TOF) *m/z*: [M + H]^+^ calcd for C_23_H_35_O_2_ 343.2632; found 343.2629. ^1^H and ^13^C NMR data for compounds **8** [18] and **9** [31] were in agreement with those previously reported.

### 3.5. Preparation of ***10*** by Methyl Ether Deprotection of ***8***

A solution of **8** (171 mg, 0.5 mmol) in dioxane (13 mL) was placed in a flame-dried flask under Ar. AgO (125 mg, 1.0 mmol) followed by 6N HNO_3_ (0.24 mL, 1.5 mmol) were added and the mixture stirred for 15 min at room temperature. Then, NaHCO_3_ (aq. sat. soln., 5 mL) was added and the mixture extracted with Et_2_O (20 mL + 2 × 5 mL). The combined organic layers were washed with H_2_O (3 × 10 mL) and brine (2 × 10 mL), dried over Na_2_SO_4_ and the solvent removed in vacuo. The crude quinone was used without purification in the next step. In this way, the residue was dissolved in CHCl_3_ (15 mL), 55 mg added of 10% Pd/C (0.025 mmol) and the flask evacuated and backfilled with H_2_ (3 cycles). After stirring the reaction mixture under an atmosphere of H_2_ (balloon) for 15 min, it was filtered through a short pad of SiO_2_ with the aid of Et_2_O (3.0 mL). Finally, the solvent was removed in vacuo and the residue purified by column chromatography (Hexane/AcOEt, 95:5) to give 138 mg of the product **10** (82%) as a white foam. IR (ATR) *v* (cm^−1^) 3375, 3082, 2925, 2873, 1541, 1490, 1192. ^1^H NMR (500 MHz, CDCl_3_) δ (ppm) 6.67 (d, *J* = 9 Hz, 1H), 6.65 (d, *J* = 3 Hz, 1H), 6.55 (dd, *J* = 9, 3 Hz, 1H), 4.91 (s, 1H), 2.93 (d, *J* = 15 Hz, 1H), 2.50 (d, *J* = 15 Hz, 1H), 2.13–2.08 (m, 1H), 2.00–1.95 (m, 1H), 1.91–1.86 (m, 2H), 1.76–1.73 (m, 1H), 1.66–1.63 (m, 1H), 1.59–1.39 (m, 5H), 1.05 (s, 3H), 1.00 (s, 3H), 0.98 (s, 3H), 0.84 (d, *J* = 7 Hz, 3H). ^13^C NMR (125 MHz, CDCl_3_) δ (ppm) 148.9 (C), 148.7 (C), 137.8 (C), 132.7 (C), 127.7 (C), 118.4 (CH), 116.5 (CH), 113.7 (CH), 41.7 (C), 40.5 (CH_2_), 39.6 (CH_2_), 39.5 (C), 35.7 (CH_2_), 34.6 (CH), 28.5 (CH_3_), 28.1 (CH_2_), 27.1 (CH_3_), 26.2 (CH_2_), 22.3 (CH_3_), 19.7 (CH_2_), 15.8 (CH_3_). HRMS (ESI/Q-TOF) *m/z*: [M + H]^+^ calcd for C_21_H_31_O_2_ 315.2319; found 315.2315. NMR data of compound **10** were consistent with those of the original isolation literature [2].

### 3.6. Synthesis of (±)-Aureol ((±)-1)

Hydroquinone **10** (157 mg, 1.0 mmol) was dissolved in anhydrous CH_2_Cl_2_ (50 mL) and the solution cooled to −60 °C. Then, BF_3_•Et_2_O (0.28 mL, 2.25 mmol) was added and the mixture stirred for 3 h at −60 °C. After that, it was warmed to –20 °C and the reaction stopped by addition of NH_4_Cl (aqueous saturated solution). The mixture was extracted with CH_2_Cl_2_ (3 × 10 mL) and the combined organic layers dried (Na_2_SO_4_) and the solvent removed in vacuo. Column chromatography of the residue (Hexane/AcOEt 9:1) yielded (±)-aureol ((±)-**1**) as a white solid (195 mg, 62%), m.p. 143–144 °C. IR (ATR) ν (cm^−1^): 3312, 3005, 3296, 2938, 2869, 1492, 1458, 1208, 948. ^1^H NMR (CDCl_3_, 500 MHz): δ 6.60 (d, *J* = 9 Hz, 1H), 6.56 (dd, *J* = 9, 3 Hz, 1H), 6.49 (d, *J* = 3 Hz, 1H), 4.26 (br s, 1H), 3.37 (d, *J* = 17 Hz, 1H), 2.11–1.99 (m, 2H), 1.97 (d, *J* = 17 Hz, 1H), 1.85–1.75 (m, 2H), 1.70–1.65 (m, 2H), 1.60–1.50 (m, 1H), 1.49–1.30 (m, 5H), 1.11 (d, *J* = 7 Hz, 3H), 1.07 (s, 3H), 0.92 (s, 3H), 0.78 (s, 3H). ^13^CNMR (CDCl_3_, 125 MHz): δ 148.3 (C), 145.8 (C), 122.2 (C), 117.3 (CH), 115.1 (CH), 114.0 (CH), 82.4 (C), 44.0 (CH), 39.3 (CH), 38.1 (C), 37.4 (CH_2_), 33.9 (CH_2_), 33.8 (C), 31.9 (CH_3_), 29.8 (CH_3_), 29.3 (CH_2_), 27.9 (CH_2_), 22.2 (CH_2_), 20.2 (CH_3_), 18.4 (CH_2_), 17.3 (CH_3_). HRMS (ESI/Q-TOF) *m/z*: [M + H]^+^ calcd for C_21_H_31_O_2_ 315.2319; found 315.2312. Physical and spectroscopic data of (±)-aureol ((±)-**1**) matched those reported in the original isolation literature [2].

## 4. Conclusions

We devised a short and efficient synthetic route for the synthesis of (±)-aureol (**1**) and (±)-5-*epi*-aureol (**11**). Our strategy relies on a C–C bond-forming reaction between (±)-albicanal (**6**) and an aryllithium derivative and a sequence of 1,2-hydride and 1,2-methyl shifts mediated by BF_3_•Et_2_O as activator and water as initiator. We are currently engaged in a computational study of the reaction mechanism, which will be published in due course. (±)-Aureol (**1**) and (±)-5-*epi*-aureol (**5**) obtained by this route are key intermediates for the synthesis of a large number of natural and synthetic derivative tetracyclic meroterpenoids, which will be used for further analysis as antitumor and antiviral agents. 

## Supplementary Materials

The following are available online at https://www.mdpi.com/1660-3397/18/9/441/s1: Figures S2–S13: ^1^H NMR of compounds **1**, **5**–**10** and ^13^C NMR of **1**, **7**–**10**.
