# Supplementary material for: A Concise Route for the Synthesis of Tetracyclic Meroterpenoids: (±)-Aureol Preparation and Mechanistic Interpretation
Source: Mar Drugs. 2020 Aug 26;18(9):441. doi: 10.3390/md18090441 (PMC7551916; doi:10.3390/md18090441)

A concise route for the synthesis of tetracyclic meroterpenoids: (±)-Aureol preparation and mechanistic interpretation.

Antonio Rosales Martínez,[1*] Lourdes Enríquez,[2] Martín Jaraíz,[2] Laura Pozo Morales,[1] Ignacio Rodríguez-García,[3] Emilio Díaz Ojeda.[1]

[1] Department of Chemical Engineering, Escuela Politécnica Superior, University of Sevilla, 41011 Sevilla, Spain
[2] Department of Electricity and Electronics, University of Valladolid, Valladolid, Spain
[3]Organic Chemistry, ceiA3, University of Almería, 04120 Almería, Spain

Correspondence: arosales@us.es

SUPPORTING INFORMATION

Table of contents

^{1}H NMR of (±)-albicanol (**5**)......S-2

^{1}H NMR of (±)-albicanal (**6**)......S-3

^{1}H NMR of **7**......S-4

^{13}C NMR of **7**......S-5

^{1}H NMR of **8**......S-6

^{13}C NMR of **8**......S-7

^{1}H NMR of **9**......S-8

^{13}C NMR of **9**......S-9

^{1}H NMR of **10**......S-10

^{13}C NMR of **10**......S-11

^{1}H NMR of (±)-aureol (**(±)-1**)......S-12

^{13}C NMR of (±)-aureol (**(±)-1**)......S-13

¹H NMR of (±)-albicanol (5)

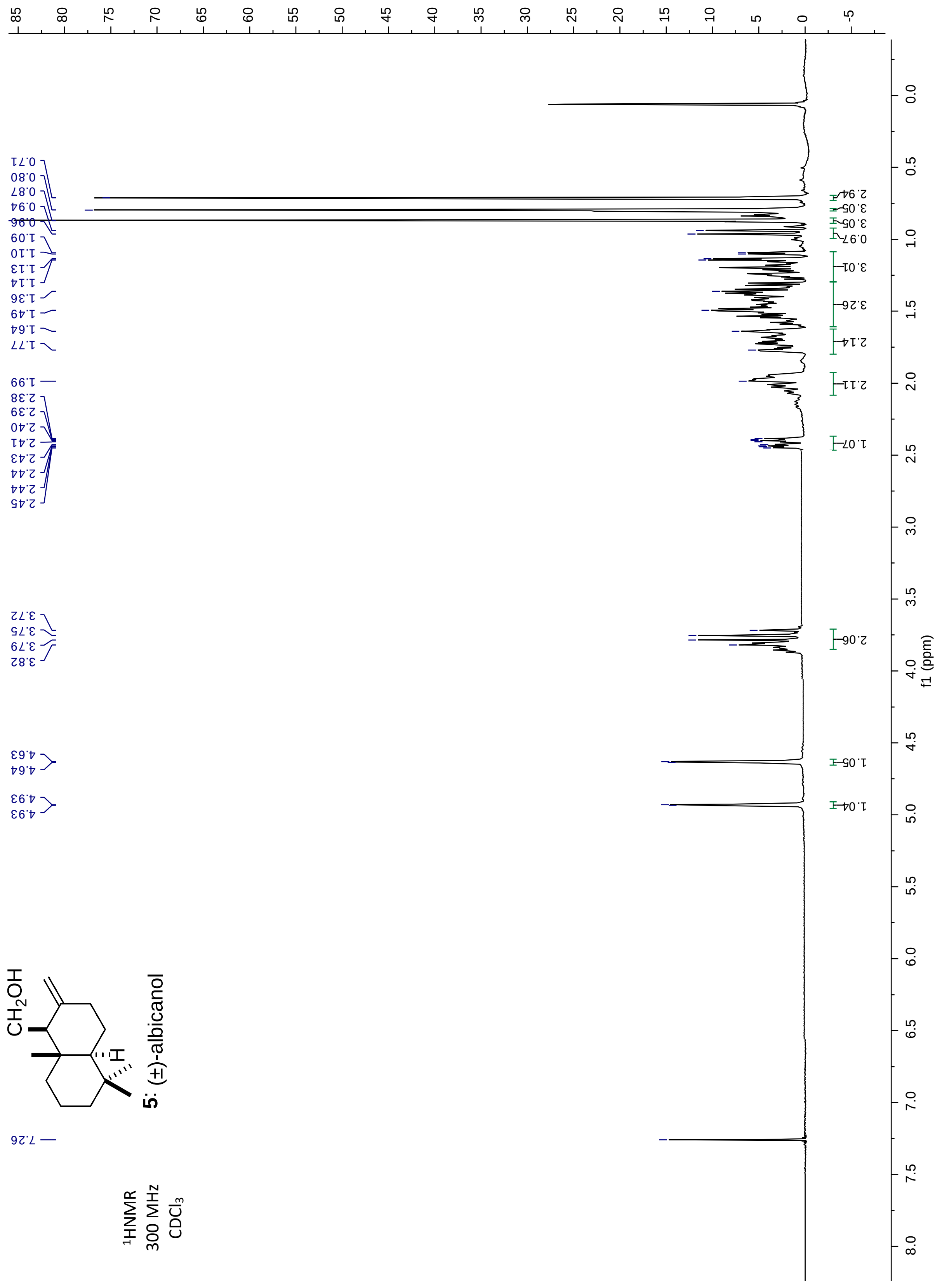

^{1}H NMR of (±)-albicanal (6)

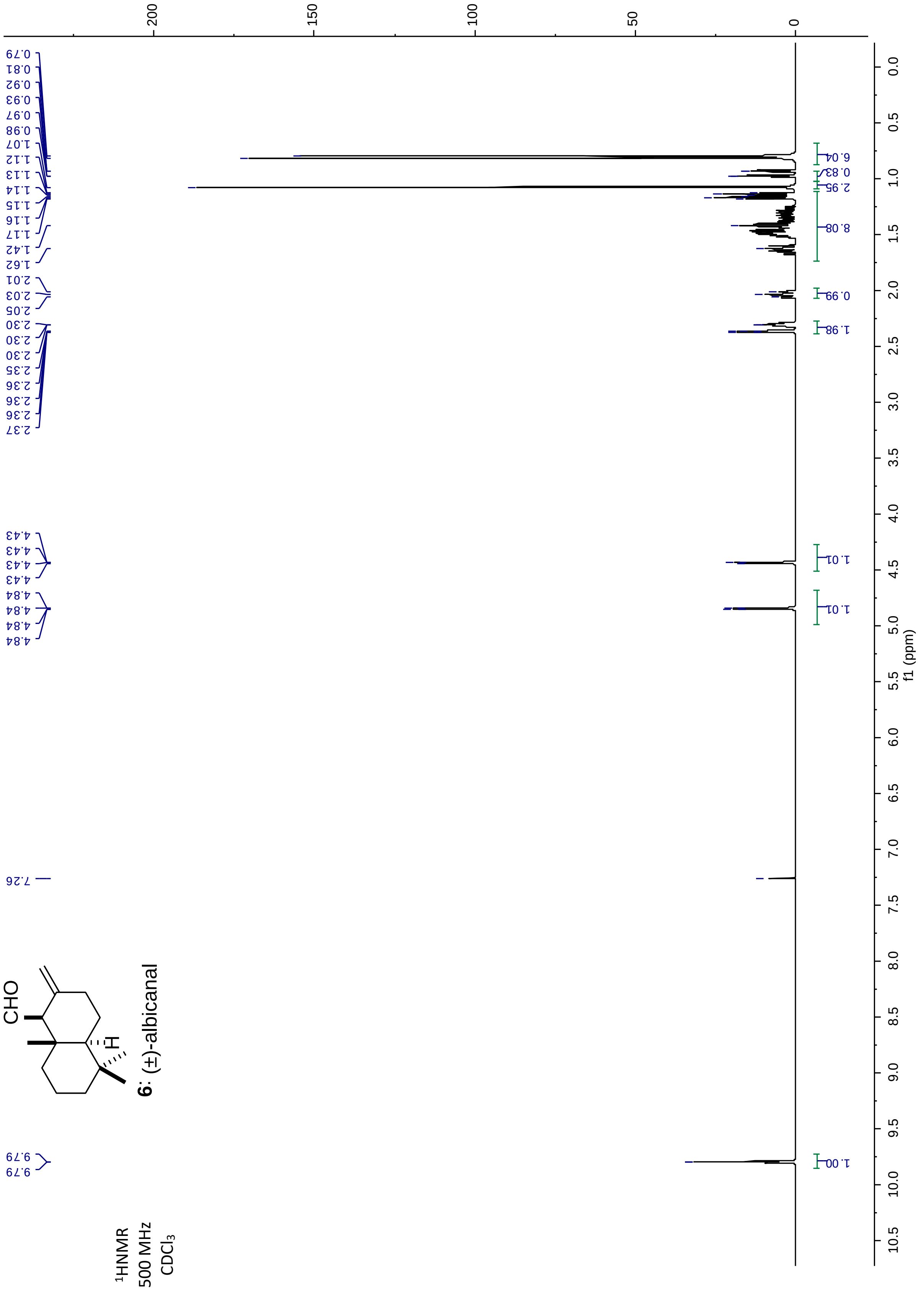

^{1}H NMR of 7

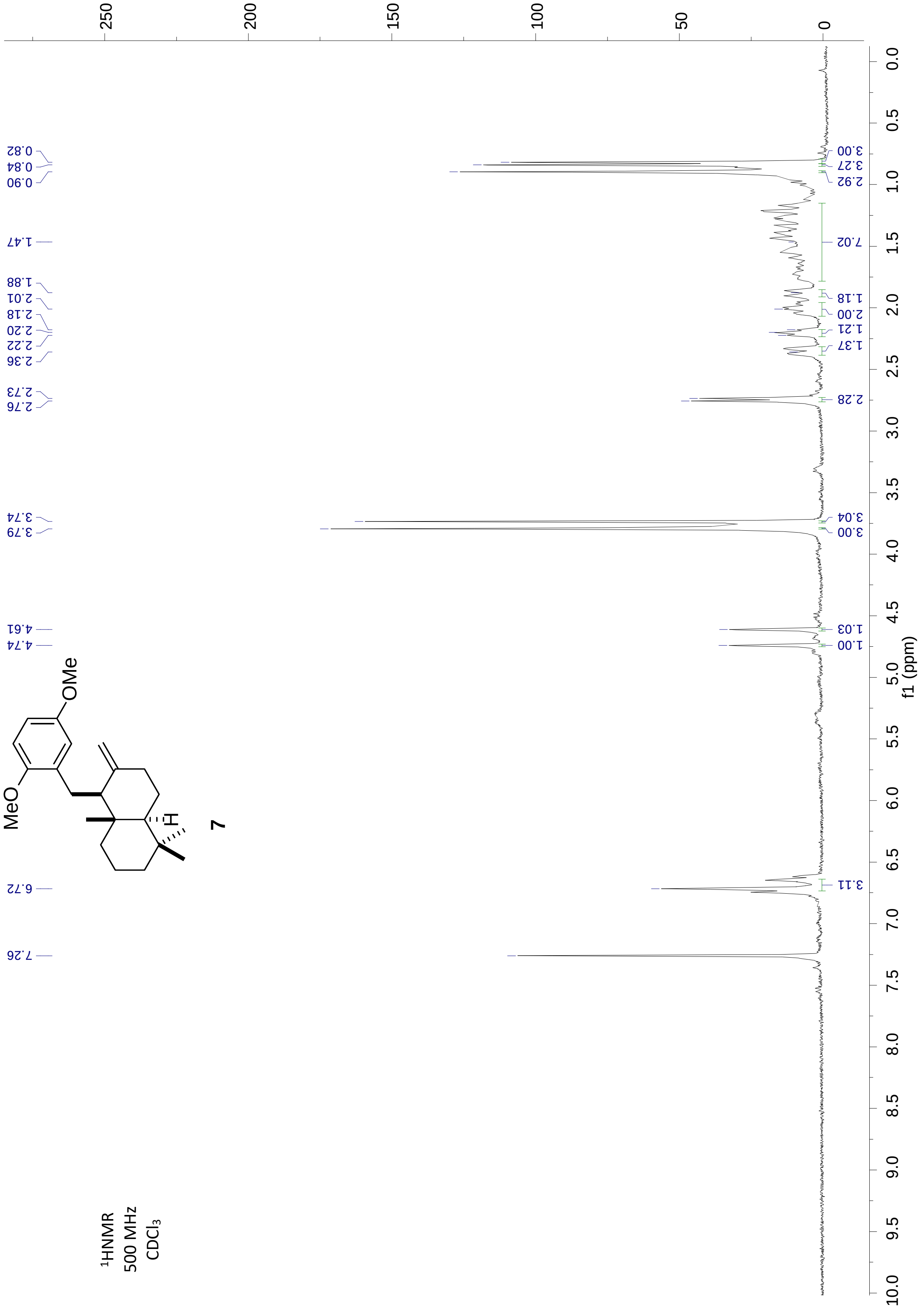

^{13}C NMR of 7

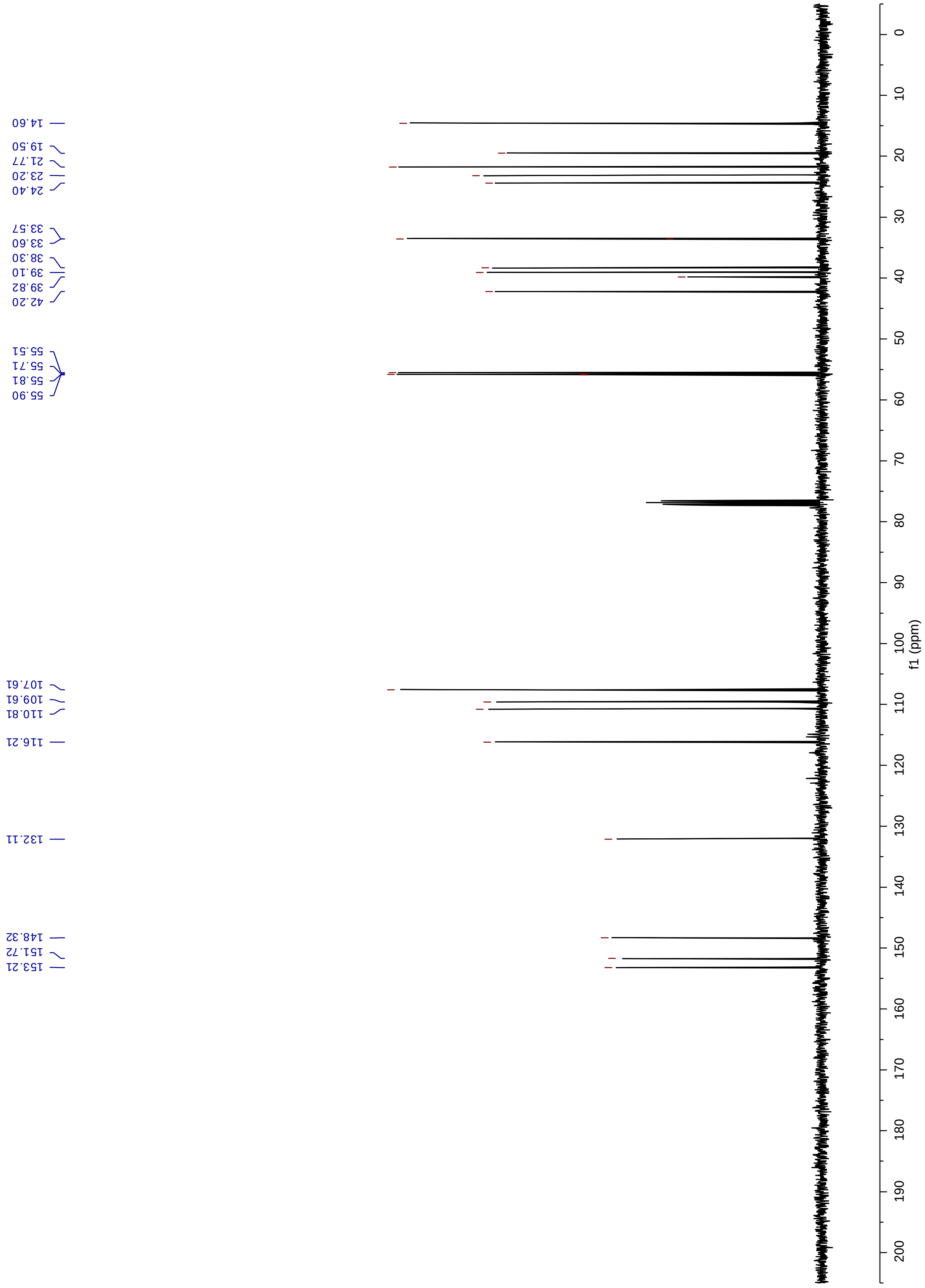
14.60
19.50
21.77
23.20
24.40
33.57
33.60
38.30
39.10
39.82
42.20
55.51
55.71
55.81
55.90
107.61
109.61
110.81
116.21
132.11
148.32
151.72
153.21
f1 (ppm)

^{1}H NMR of 8

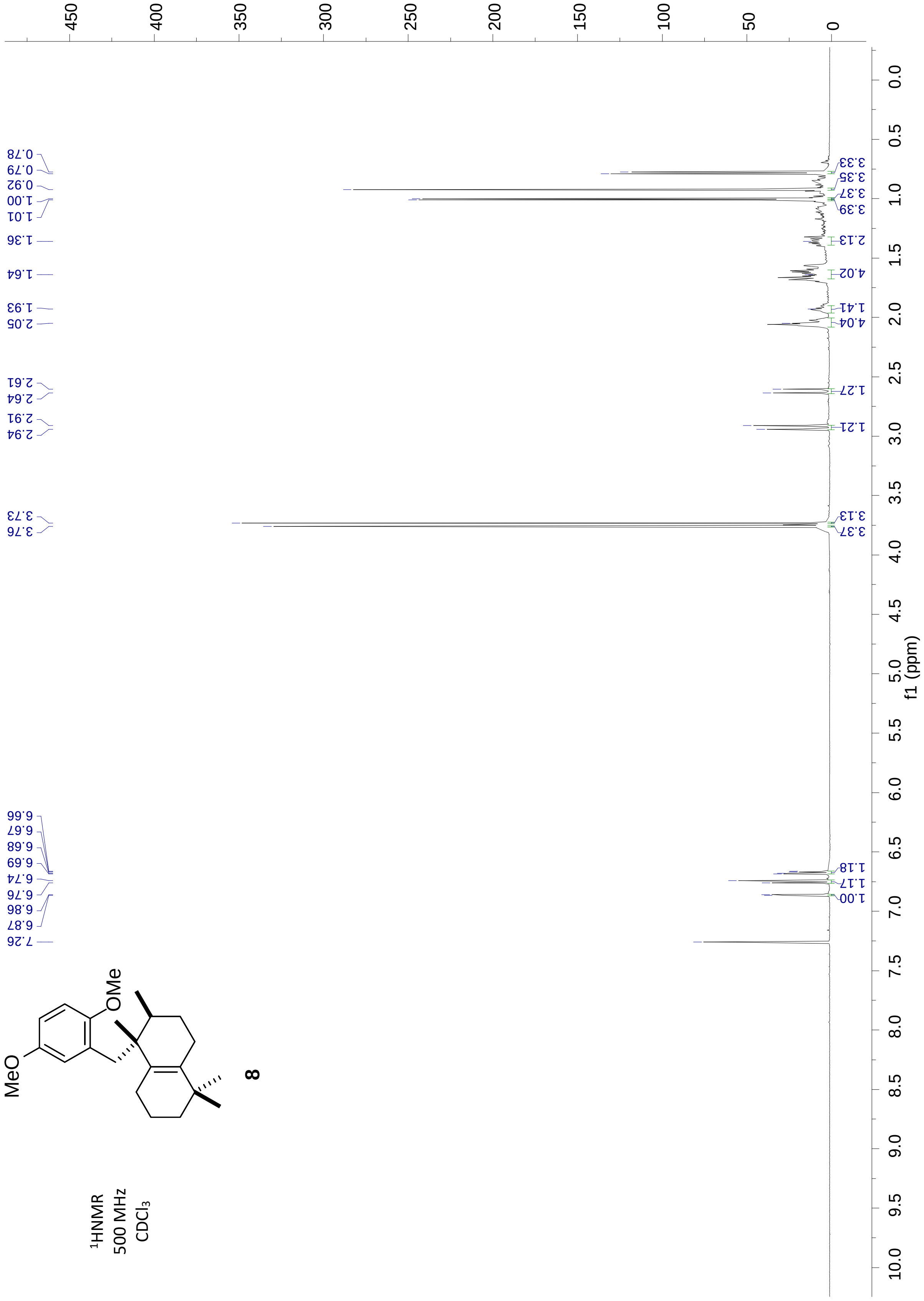

^{13}C NMR of 8

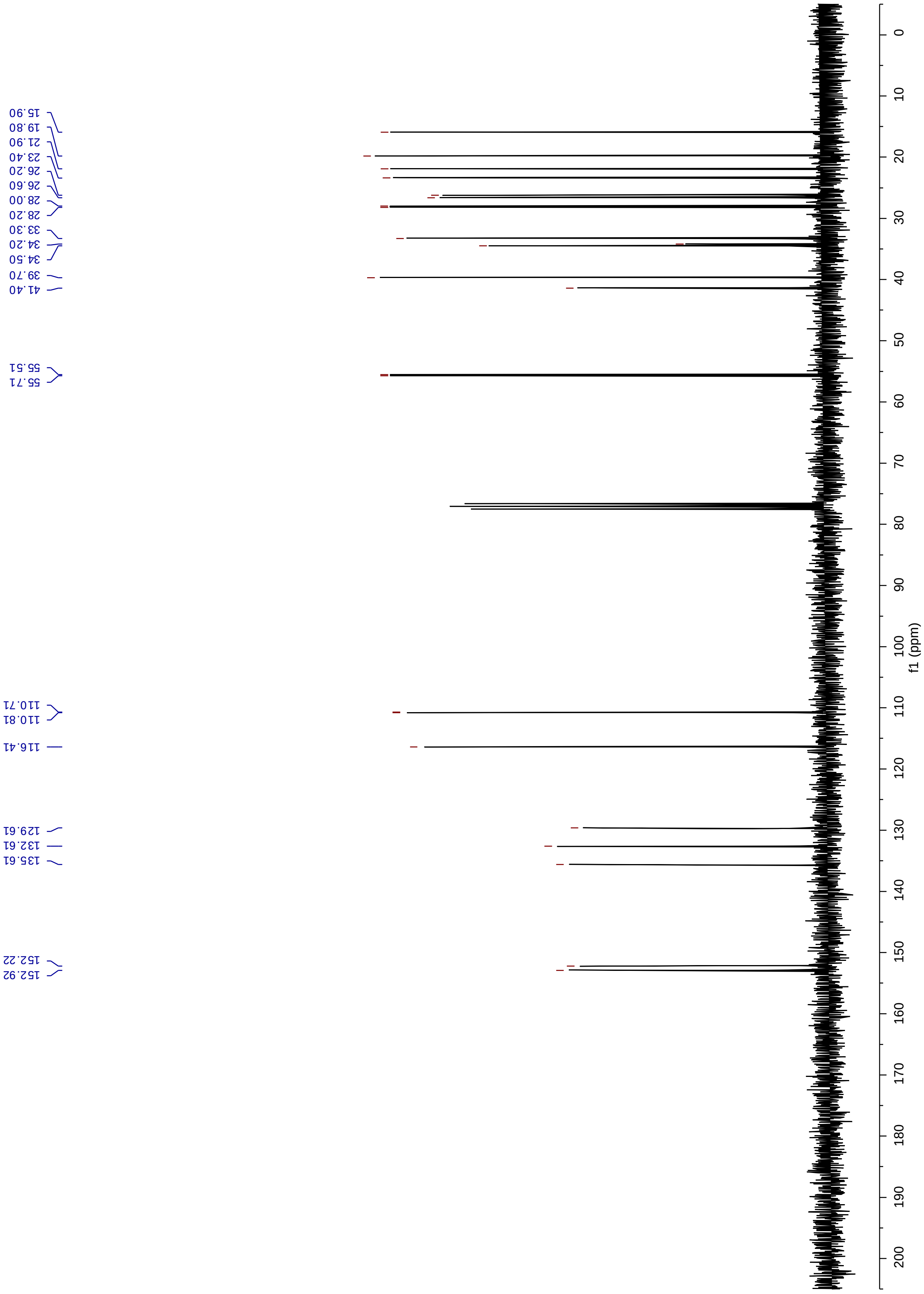

15.90
19.80
21.90
23.40
26.20
26.60
28.00
28.20
33.30
34.20
34.50
39.70
41.40
55.51
55.71
110.71
110.81
116.41
129.61
132.61
135.61
152.22
152.92
f1 (ppm)

^{1}H NMR of 9

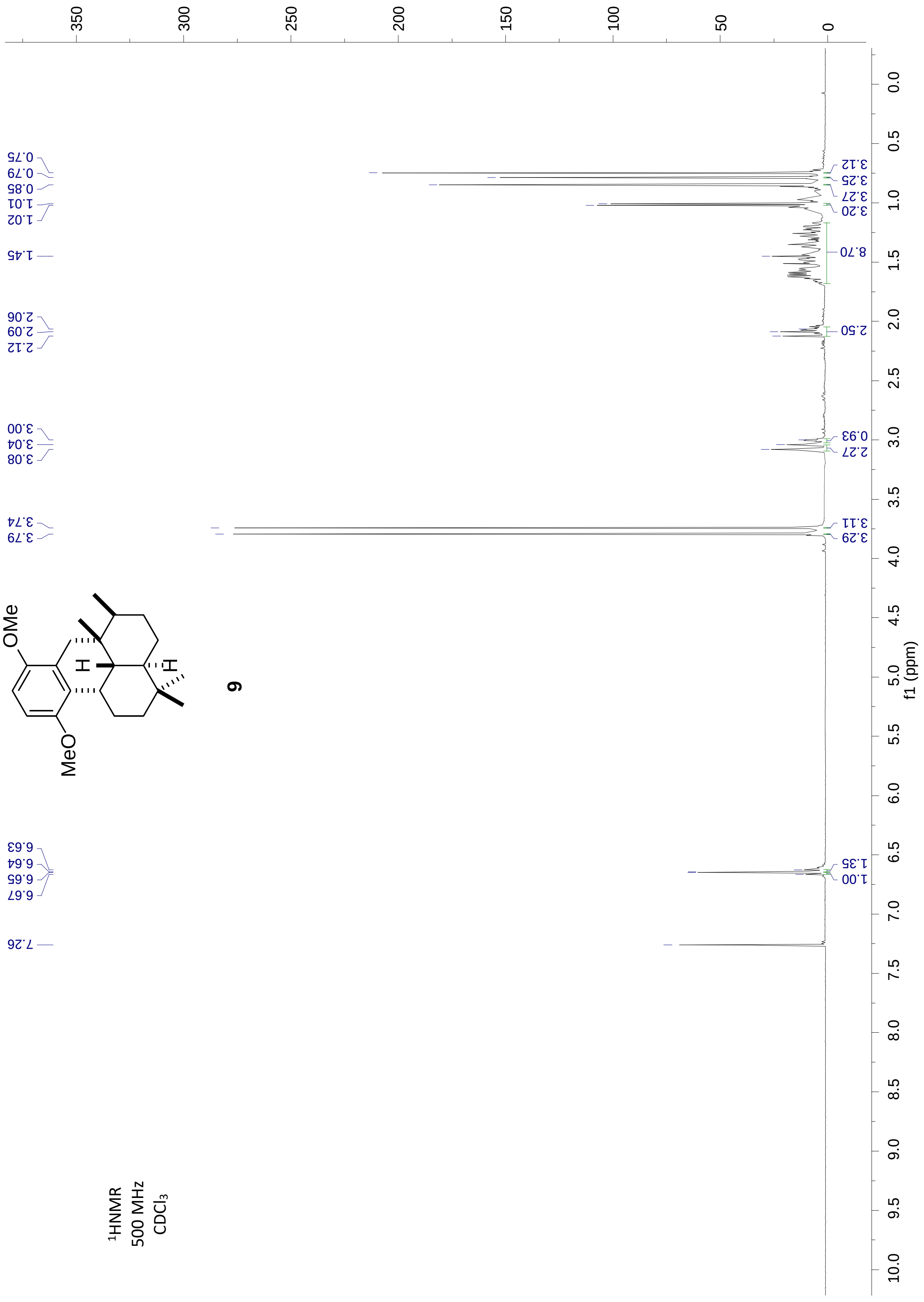

^{13}C NMR of 9

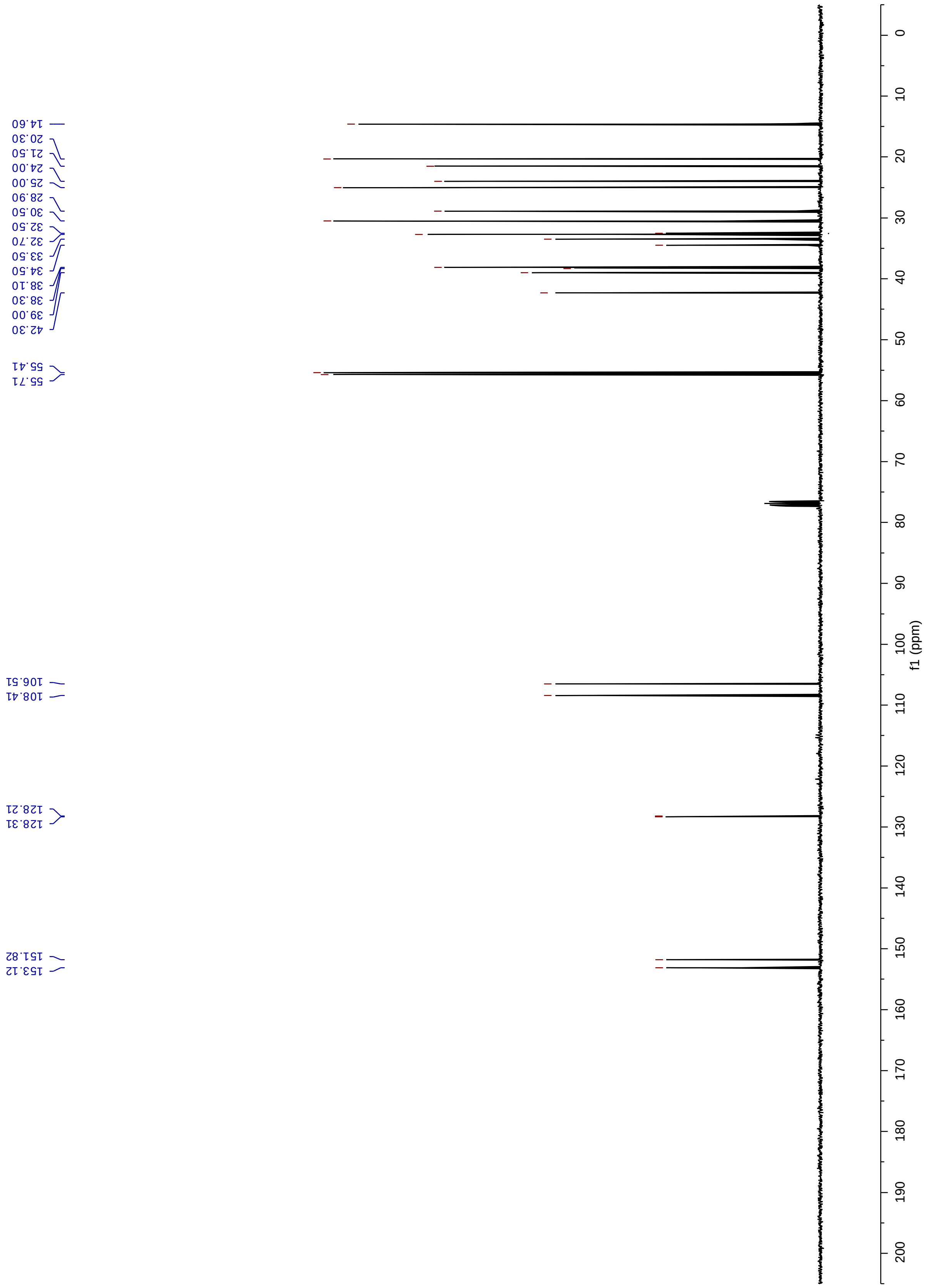
14.60
20.30
21.50
24.00
25.00
28.90
30.50
32.50
32.70
33.50
34.50
38.10
38.30
39.00
42.30
55.41
55.71
106.51
108.41
128.21
128.31
151.82
153.12
f1 (ppm)

^{1}H NMR of 10

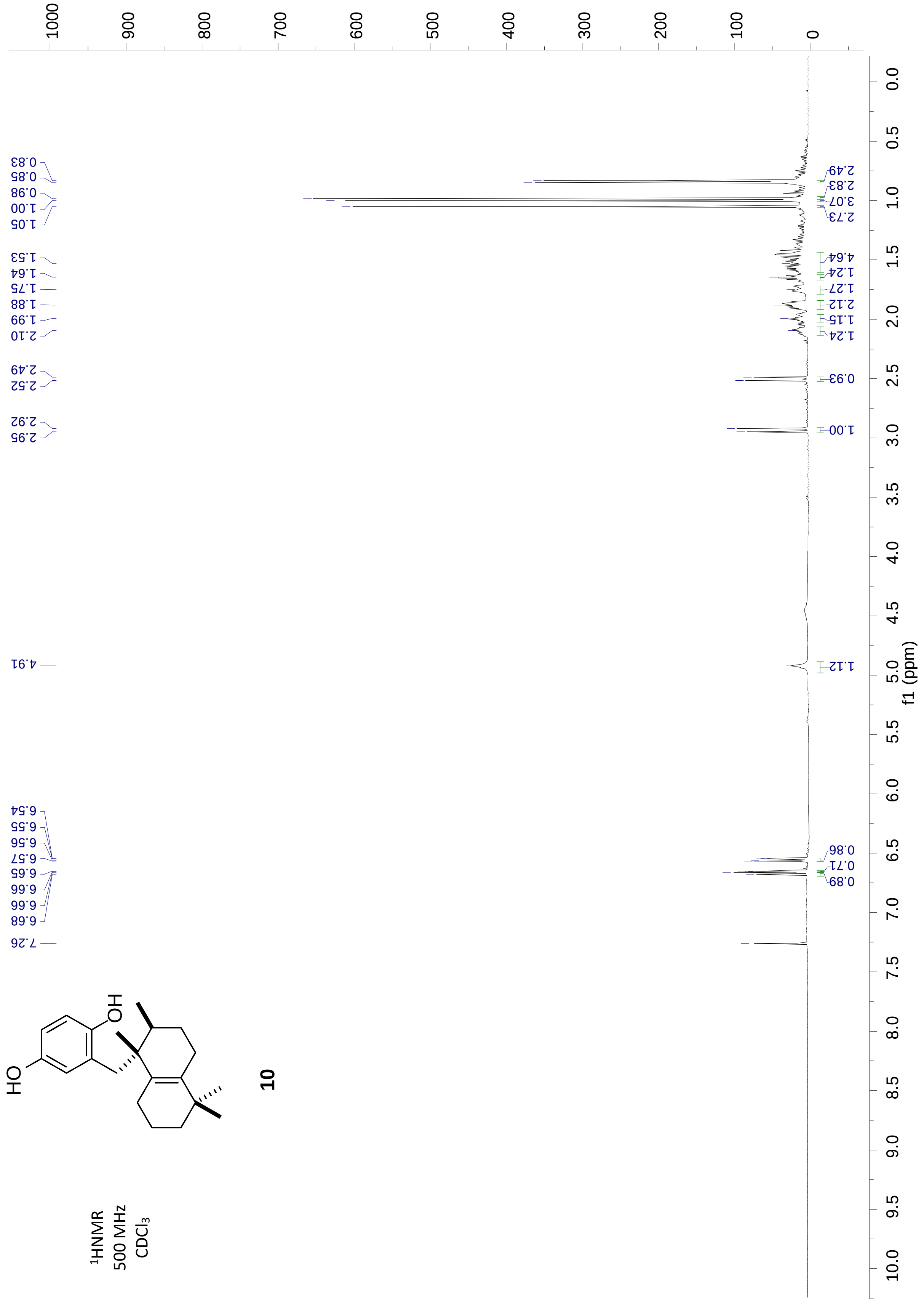

^{13}C NMR of 10

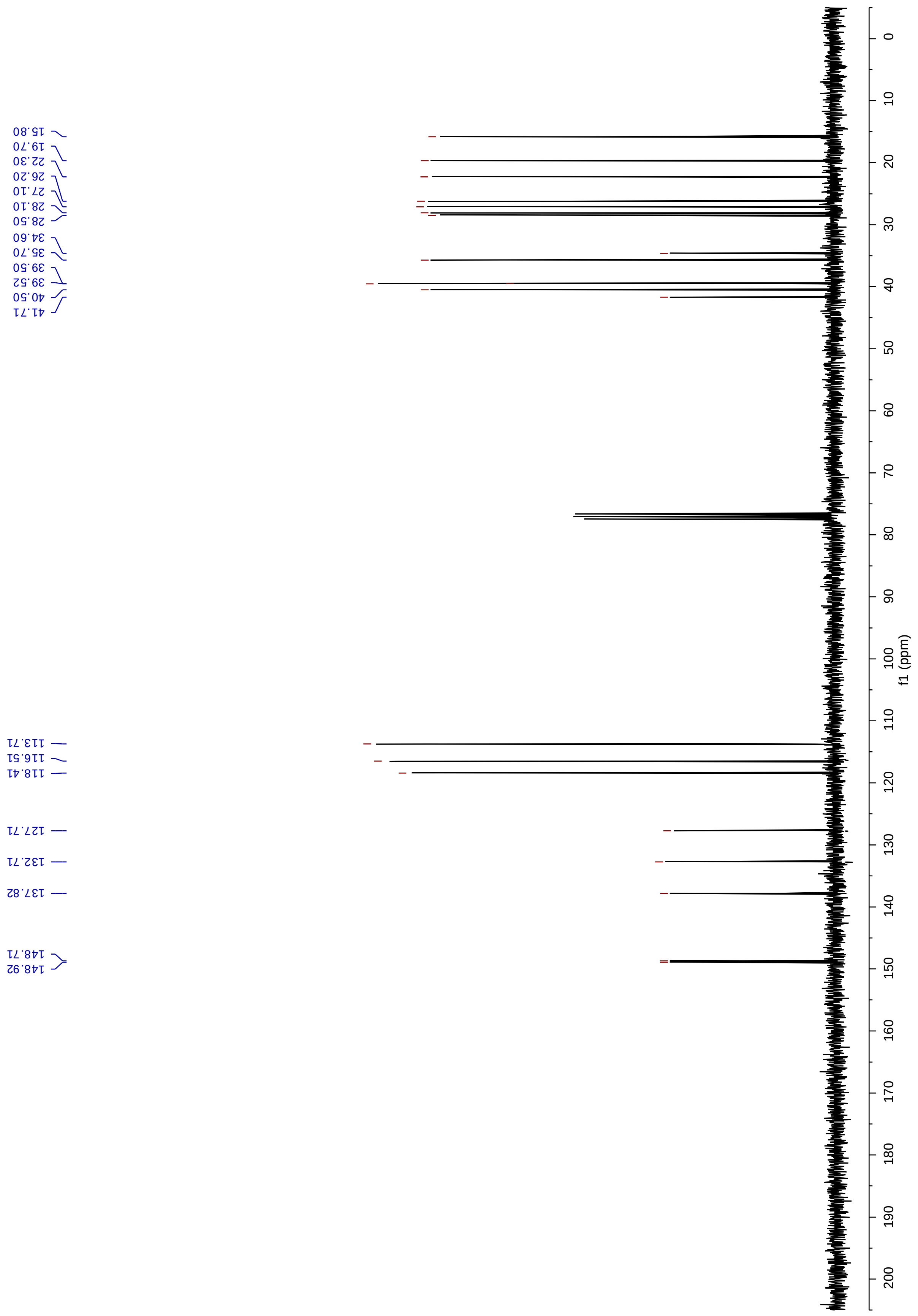
15.80
19.70
22.30
26.20
27.10
28.10
28.50
34.60
35.70
39.50
39.52
40.50
41.71
113.71
116.51
118.41
127.71
132.71
137.82
148.71
148.92
f1 (ppm)

^{1}H NMR of (±)-aureol ((±)-1)

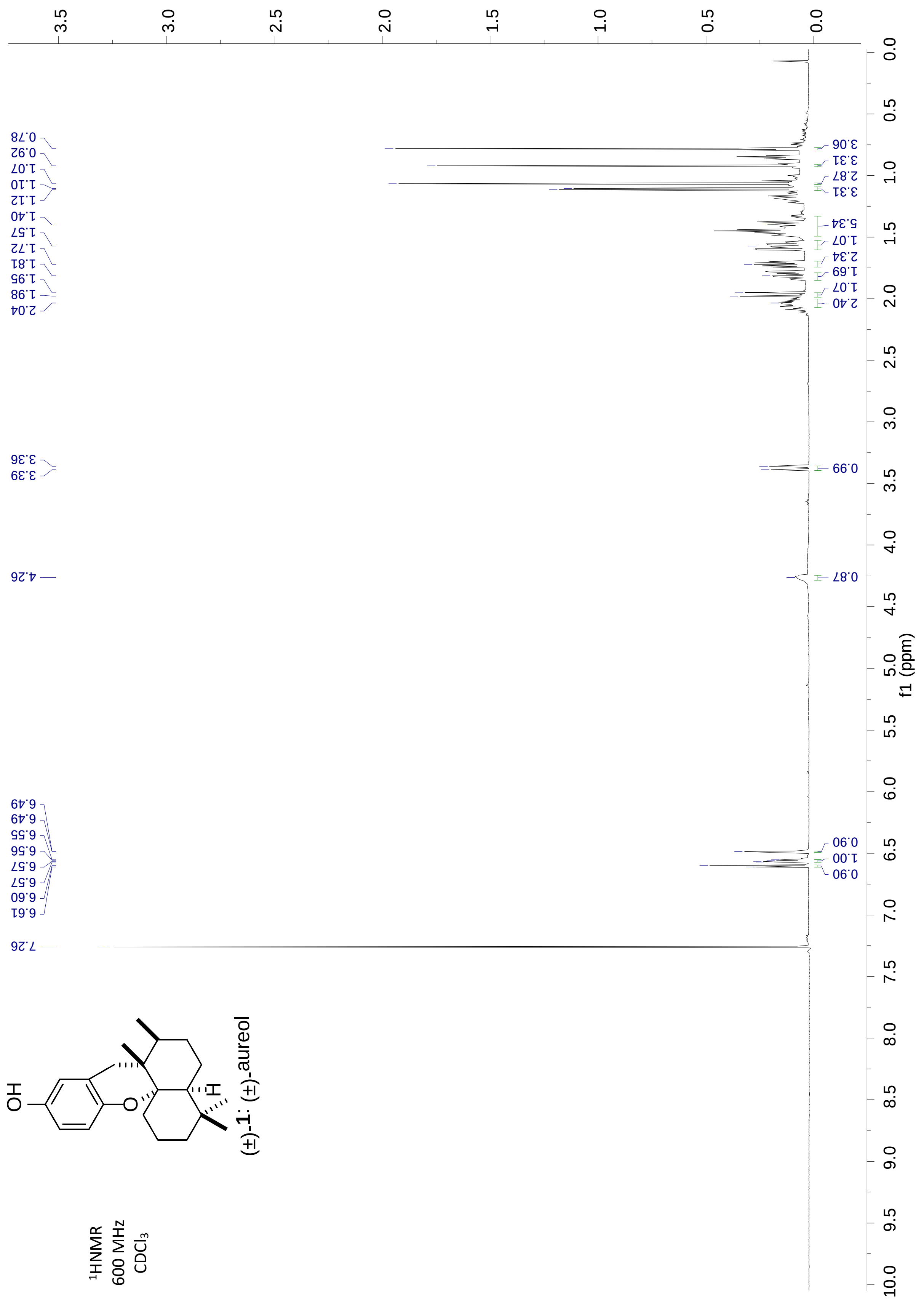

^{13}C NMR of (±)-aureol ((±)-1)

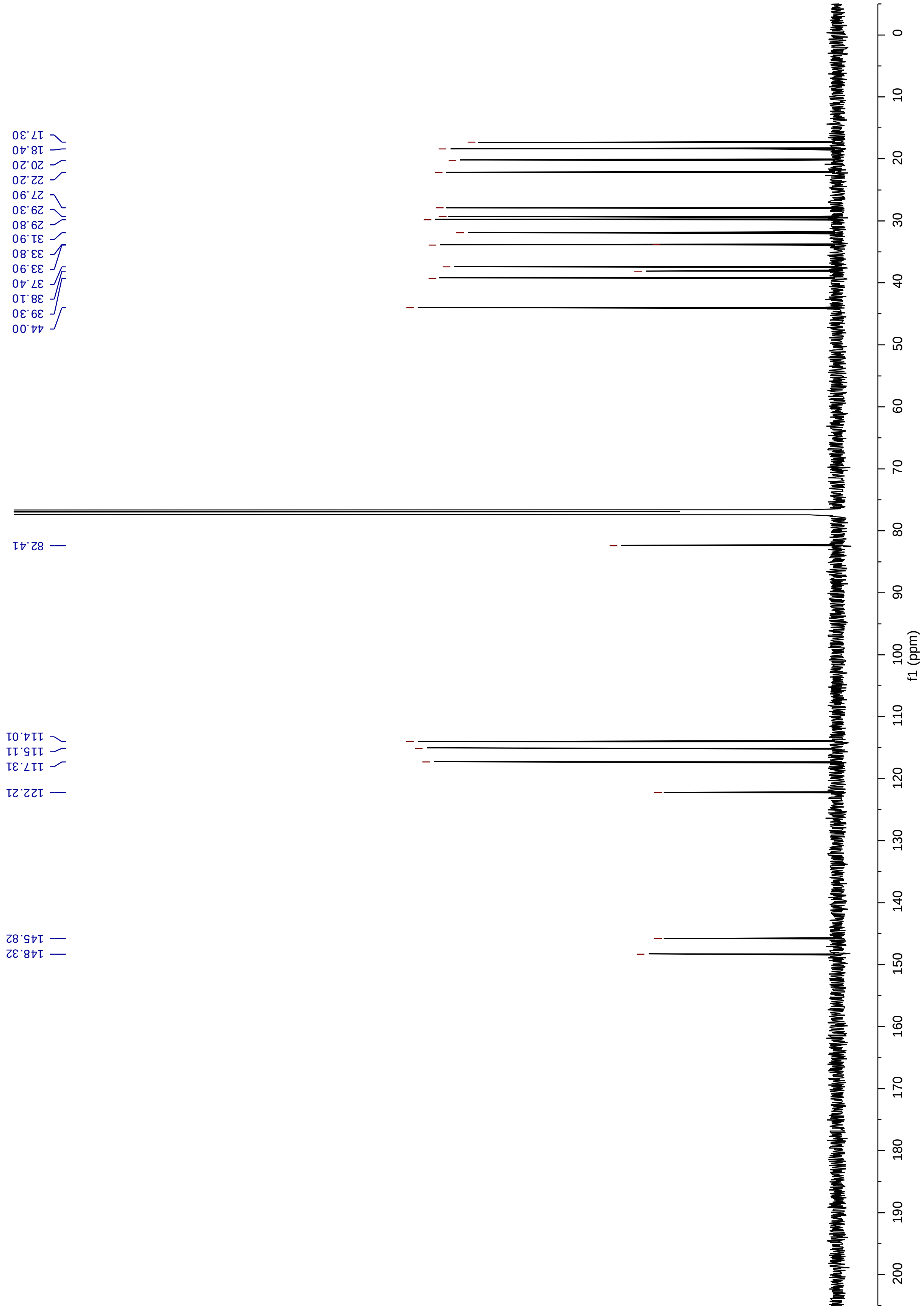

Supplement: Supplementary file 1 [file marinedrugs-18-00441-s001.pdf]
